# Knowledge, attitudes and practices on Schistosomiasis in sub-Saharan Africa: a systematic review

**DOI:** 10.1186/s12879-017-2923-6

**Published:** 2018-01-18

**Authors:** Hlengiwe Sacolo, Moses Chimbari, Chester Kalinda

**Affiliations:** 1 School of Nursing and Public Health, College of Health Sciences, University of KwaZulu-Natal, Howard Campus, Durban, South Africa; 2 College of Health Sciences, University of KwaZulu-Natal, Howard Campus, Durban, South Africa

**Keywords:** Schistosomiasis, *S. mansoni*, S. Haematobium, Knowledge, Attitudes, Perceptions, Beliefs, Practices, Sub-Saharan Africa

## Abstract

**Background:**

Schistosomiasis remains a global health problem with an estimated 250 million people in 78 countries infected, of whom 85% live in Sub-Saharan Africa. Preventive chemotherapy remains the key public health strategy to combat schistosomiasis worldwide. Recently the WHO emphasized on the use of integrative approaches in the control and elimination of schistosomiasis. However, a detailed understanding of sociocultural factors that may influence the uptake of the intended health activities and services is vital. Thus, our study sought to understand the knowledge, attitudes, perceptions, beliefs and practices about schistosomiasis in various communities in Sub-Saharan Africa.

**Methods:**

A systematic search of literature for the period 2006–2016 was done on Medline, PubMed, CINAHL, Psych info and Google Scholar using the following key words “*Schistosomiasis, S. mansoni, S. haematobium, knowledge, attitudes, perceptions, beliefs and practices in Sub-Saharan Africa*” in combination with Bolean operators (OR, AND). In this context, we reviewed studies conducted among school children, community members and caregivers of preschool children. Thematic analysis was utilised for the overall synthesis of the selected studies. This was done after reading the articles in depth. Themes were identified and examined for similarities, differences and contradictions.

**Results:**

Gaps in schistosomiasis related knowledge and sociocultural barriers towards the uptake of preventive and treatment services among communities in Sub-Saharan Africa were identified. In addition to limited knowledge and negative attitudes, risky water related practices among community members, school children and caregivers of preschool children were identified as key factors promoting transmission of the disease.

**Conclusion:**

The study concluded that a comprehensive health education programme using contextual and standardised training tools may improve peoples’ knowledge, attitudes and practices in relation to schistosomiasis prevention and control. Findings also highlight the significance of including caregivers in the planning and implementation schistosomiasis control programs targeting pre-school children.

**Electronic supplementary material:**

The online version of this article (10.1186/s12879-017-2923-6) contains supplementary material, which is available to authorized users.

## Background

Schistosomiasis, widely known as bilharzia, remains a public health problem in several parts of the world, particularly in Africa. Despite numerous programs aimed at combatting the global prevalence, infection rates remain high, particularly in sub Saharan Africa which accounts for over 85% of people living with schistosomiasis in a population that only constitutes 13% of the world’s population [1]. Worldwide, there are six species of schistosomes that affect human beings [2]. *Schistosoma intercalatum, Schistosoma mekongi*, *Schistosoma japonicum* and *Schistosoma guineesis* are localized to specific settings whilst, *Schistosoma haematobium* and *Schistosoma mansoni* are wide spread [3]. Of all these species, the most prevalent in Sub-Saharan Africa are *S. haematobium* and *S. mansoni* which cause urogenital and intestinal schistosomiasis, respectively [4].

The scale and trend of the disease burden in Sub-Saharan Africa varies considerably, with poverty stricken and marginalized communities being the most affected [5]. These populations have low socio-economic status with limited access to clean water and adequate sanitation. The perpetual progression of schistosomiasis in this region is coupled with devastating and long-lasting effects. For instance, even though schistosomiasis is preventable and treatable, if left untreated it can lead to debilitating and irreversible clinical complications such as liver and spleen enlargement, bladder ulceration and deformities, infertility and kidney blockage [6, 7]. Across Sub-Saharan Africa, children, women and those working in contact with natural water bodies continue to be at greater risk [8, 9]. Researchers suggest that success in schistosomiasis prevention among these groups has the potential to reduce the rate of transmission among the general population and ultimately reduce the population prevalence of schistosomiasis [10]. The increased risk of infection among such groups is true for all countries in Sub-Saharan Africa and is attributed mainly to risky water practices, poor sanitation, lack of knowledge, negative attitudes and beliefs about schistosomiasis. Among women, low educational status has been found to be an additional predictor of infection [11].

Another explanation for the heightened prevalence of schistosomiasis can be drawn from recent findings pointing to the exclusion of under-fives from MDA programs and subsequently care givers from health education campaigns in endemic areas [12, 13]. Most schistosomiasis educational programs target school going children because they are easy to reach [14]. This however excludes community members including caregivers who may unknowingly predispose preschool children entrusted to their care to schistosomiasis infection. The positive influence of comprehensive knowledge, positive attitudes and practices on effective prevention and control of parasitic infections is well established [15–17]. The degree of such influence however varies according to community settings and programs implemented. This highlights the need for relevant and vibrant health education programs addressing sociocultural barriers towards sustained schistosomiasis prevention and control in the African context.

Previous WHO recommendations mainly supported the regular treatment of at least 75% of school aged children at risk as basis of control [18]. That eventually resulted in the skewed focus on funding, schistosomiasis control programs and research on school going children aged 6–15 years old whilst depriving the younger and older ones of such benefits. In 2012 however, the World Health Assembly adopted resolution (WHA 65.21) that called for the elimination of schistosomiasis through the use of integrated control strategies inclusive of all vulnerable groups [8]. This resolution seems to be in line the Supply–Enabling Environment–Demand (SEED) programming model [19] which asserts that disease elimination may be possible through simultaneous implementation of the following tasks: (i) provision of timely treatment and quality service delivery, (ii) promotion of positive health seeking behaviour, improved knowledge, attitudes and practices that ensure sustainability of control efforts; and (iii) provision of an enabling environment for effective service delivery that includes the establishment of national policies, guidelines, programs and a community environment that promotes disease elimination. The Supply–Enabling Environment–Demand (SEED) programming model stems from the law of supply and demand and the enabling environment theory which have been used by numerous studies across disciplines, population groups and countries [20–25]. Given the broadness in scope of the key concepts used in the model and its consideration of social norms and behaviour change, it is easily adapted to give guidance to programs in various fields of study. It has been used recently among children to guide child protection programs and to generate evidence on the extent to which children with disability access school education in eastern and Southern Africa [26].

The WHO encourages countries to adopt policies and programs focusing on eliminating schistosomiasis rather than just controlling its morbidity, thus the need to integrate MDA programs with primary health care interventions such as effective health education programs [18]. Essentially, health education should be the heartbeat of all health promoting interventions in order to yield sustainable positive changes. Designing such programs however warrants the understanding of existing knowledge, attitudes, perceptions, beliefs and practices about schistosomiasis in endemic communities. Our review therefore aimed at understanding the role of knowledge, attitudes and practices (KAP) in relation to schistosomiasis prevention and control in various communities in Sub-Saharan Africa. The review contributes to knowledge that will influence policy decisions in Southern Africa regarding the inclusion of under-five children in schistosomiasis treatment programs.

## Methods

### Searching strategy

We conducted a systematic review of literature using articles published between 2006 and 2016 because we wanted to assess what issues have been raised in the past 10 years so as to identify key research gaps for the work we intent carrying out on the subject matter. The literature search was done independently by two investigators (HS, CK) who searched for articles in various databases (Medline, PubMed, CINAHL, Psych info and Google Scholar), using the following key words “*Schistosomiasis, S. mansoni, S. haematobium, knowledge, attitudes, perceptions, beliefs, and practices in Sub-Saharan Africa*”. Search terms used in this review were initially broad in scope in an attempt to cover a wide breadth of knowledge in answering the research question. After this, key word synonyms were identified and combined with key words to allow a more comprehensive search. The search used both ‘free terms’ and ‘index terms’ funnelled using the Bolean operators (OR, AND) and truncations. Our search was limited to articles published in English. We followed the PRISMA (Preferred Reporting Items for Systematic Reviews and Meta-Analyses) guidelines for conducting and reporting this systematic review.

### Study selection

**Fig. 1:**
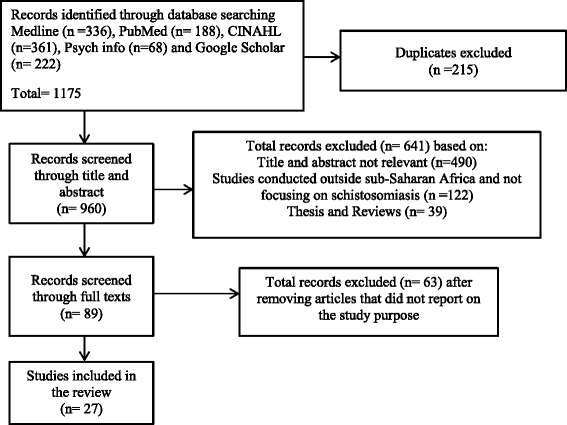
PRISMA flow diagram

The review strategy was carried out in three stages. The first stage was identification of records through data base searching; the second stage involved the screening of abstracts by two authors in order to carefully select literature that met the inclusion criteria and the final stage was assessment for eligibility. The process followed is illustrated in the PRISMA flow diagram (Figure 1). Twenty seven full articles were deemed eligible for the review. Studies were included if they were published between 2006 and 2016, conducted in countries within Sub-Saharan Africa provided they focused on awareness, knowledge, attitudes, perceptions, beliefs and practices on schistosomiasis. Papers selected were scrutinised to find the best available evidences to support the study purpose.

### Quality assessment

Quality appraisal was assessed using a critical appraisal tool for assessment of cross-sectional studies modified from Downes, Brennan [27]. The total quality score was computed for each study using the following indicators which were also in line with the study objectives: (1) clear definition of objectives and aims; (2) study design appropriate for the stated aims; (3) sample size justified; (4) target population clearly defined (appropriate population base/unbiased sampling) (5) risk factor and outcome variables measured correctly using instruments that had been trialled, piloted or published previously; (6) methods (including statistical methods) sufficiently described to enable them to be repeated; (7) results for analysis described in the methods presented; (8) authors discussions and conclusions justified by results; (9) limitations of the study discussed; (10) ethical approval or consent of participants attained. Answers were scored 0 and 1 for ‘No’ and ‘Yes’, respectively. Total scores varied between 0 and 10 where 1–4 = (Low); 5–7 = (Moderate) and 8–10 = (High). The total quality scores in this study ranged from moderate to high across studies (Additional file 1).

### Data abstraction

For studies that were identified and selected for inclusion in the review, we extracted information about the study: first author, year of publication, study objectives, study population, study location and summary of the findings. Studies were presented in three categories; those conducted among (1) community members, (2) caregivers and (3) school children. Thematic analysis was utilised for the overall synthesis of the selected studies. This was done after reading the articles in depth. Themes were identified and examined for similarities, differences and contradictions.

## Results

For this study, 1175 articles studies were initially identified from the five data bases. The articles were screened and 960 were retained after 215 duplicated articles were removed. Of those articles, 641 were excluded for the following reasons (1) title and abstract not relevant (2) not conducted in Sub-Saharan Africa (3) did not focus exclusively on schistosomiasis (4) were not peer reviewed articles. Only 89 articles were considered for full text reviewing. Out of these, 27 were retained and included in this review. The primary evidence base for these studies comprised of quantitative, qualitative and mixed method studies. Thematic analysis was utilised for the overall synthesis of the selected studies. This was done after reading the articles in depth. Themes were identified and examined for similarities, differences and contradictions.

Four major themes emerged from this review: (1) socio-demographic (2) knowledge and awareness on schistosomiasis (3) attitudes and beliefs related to schistosomiasis (4) Practices related to schistosomiasis prevention and control. The review also displays three categories of KAP studies. There were 18 community based KAP studies, 4 school based studies and 5 conducted among care givers of children aged 5 years and below. The studies synthesised in this review are from nine Sub-Saharan countries namely: Kenya (*n* = 5), Uganda (*n* = 4), Nigeria (*n* = 6), Mozambique (*n* = 1), Ghana (*n* = 3), Tanzania (n = 5), Malawi (n = 1), South Africa (n = 1) and Swaziland (n = 1). Table 1 summarises the papers.

**Table 1:** Summary of studies that were used in the scoping Review

Author/Year	Study objectives	Type of study	Population/study location	Summary of main findings
Community based KAP studies (*n* = 18)				
Adoka et al. [39] / 2014	To assess the community’s knowledge and perceptions of schistosomiasis prevalence, transmission and control in relation to aquatic habitats in the Lake Victoria basin of Kenya.	Cross-sectional study using semi-structured questionnaires	243 community members/ Lake Victoria basin of Kenya.	Sociodemographic variables: knowledge was associated with educational level, being male and occupation type Knowledge: (i) 42% of respondents had no idea on how schistosomiasis is contracted, (ii) 22% rightly mentioned causes of schistosomiasis. (iii) Only 3% of the respondents were familiar with the life cycle of schistosomiasis and (iv) 38% had no idea how schistosomiasis is treated. *Misconceptions:* 18% mentioned drinking/eating dirty water/food was the cause for schistosomiasis. Practices: Risky water practices included, harvesting hippo grass, fishing, and washing clothes and utensils and bathing in rivers infested with intermediate host snails
Anguza et al. [28] / 2007	To elicit and understand peoples’ perceptions of intestinal schistosomiasis that is a prerequisite for designing appropriate control strategies.	Mixed method study using 6 FGDs and 432 semi-structured interviews	Community members (64 FGD participants and 432 respondents)/ Busia district of Uganda	Sociodemographic variables: knowledge and practices were associated with being male, educated and employed Knowledge: (i) 97% were aware of schistosomiasis, (ii) 14% were able to mention symptoms however early symptoms were poorly understood by all participants and (iii) only 45% knew about the existence of a schistosomiasis control program through peers and health workers *Misconceptions:* 78% said schistosomiasis can be prevented by not drinking contaminated water. Attitudes: Respondents perceived community involvement in schistosomiasis control to be very low, mainly due to fear of side effects Practices: (i) Traditional medicine was preferred for schistosomiasis treatment. (ii) About 70% of people practiced risky water practices whilst 88% admitted that people defecate in bushes and in the lake due to lack of latrines.
Adeneye et al. [17] / 2007	To describe sociocultural factors that influences the distribution process of praziquantel for the mass treatment of schistosomiasis infection.	Qualitative study: FGDs and in-depth interviews were held with adolescents, children and Adults from six communities before and after MDA program implementation	Adults, adolescents and children / Ogun State, Southwest Nigeria	Knowledge: There was a high level of awareness on schistosomiasis; however causes of infection were poorly understood. *Misconceptions:* Community members believed that schistosomiasis was caused by urinating at a T-junction on the road. Attitudes: People thought treatment was expensive and doubted its efficacy prior to MDA Practices: (i) Participants became more receptive of treatment after program due to perceived efficacy of praziquantel against the disease and its availability. (ii) Community based interventions had more successful treatment coverage compared to the Primary Health Care-centred or school based approaches
Dawaki et al. [29] / 2015	To evaluate the knowledge, attitude and practices (KAP) regarding schistosomiasis among rural Hausa communities in Kano State, Nigeria.	Cross-sectional study using a structured questionnaire	551 individuals from rural communities in Kano State in Nigeria	Sociodemographic variables: Males, those educated or employed and younger respondents were more knowledgeable compared to their counterparts. Knowledge: (i) 74.5% were aware of urinary schistosomiasis; major sources of information were family and neighbours (ii) 38.6% of participants were ignorant of signs and symptoms, (iii) 67.0% had no knowledge on transmission and (iv) 63.8% were ignorant of all preventive measures. *Misconception:* 18.9% of the respondents believed that schistosomiasis is caused by eating salty or sour food or that it can be spread by sharing a toilet with an infected person Attitudes: Three-quarters of the respondents considered schistosomiasis a serious disease. Practices: (i) Children and male adolescents were observed bathing/swimming in streams and ponds and human excreta was seen around water bodies and within farmlands. (ii) Only 34.7% of the participants sought treatment from clinics/hospitals, almost 50% practiced self-medication and 15.3% either used traditional medicine or nothing.
Fleming et al. [40] / 2009	To describe the perceptions, attitudes, constraints and experiences of those implementing the programme and recipients of treatment.	Qualitative study in 20 districts implementing the programme	Community members/ *L. Victoria* islands in Uganda.	Attitudes: MDA was perceived to be beneficial because it improved participants health conditions, Practices: Poor health seeking behaviour was related to side-effects, smell and size of praziquantel (PZQ), together with shortage of Praziquantel in health facilities
Kabatereine et al. [16] / 2014	To assess community awareness on schistosomiasis KAP	Cross sectional descriptive study using a semi structured questionnaire	908 household heads, 286 drug distributors, 181 pupils,104 teachers, 47 biomedical workers/ Lake Victoria, Uganda	Socio-demographic variables: Males were 1.5 times more knowledgeable compared to females. Tertiary education, treatment history and staying longer in islands were also determinants of higher knowledge. Knowledge: Biomedical staff (92.3%), pupils (84.3%), teachers (80.4%) and household heads (87.3%) knew about schistosomiasis. However knowledge on transmission was poor as shown by, 38% and 50% biomedical staff and household heads respectively. *Misconceptions:* Fifteen percent of household heads believed schistosomiasis was caused by drinking dirty water and eating contaminated food Practices: (i) Open defaecation was very common, only 33% house hold heads had latrines in their homes. (ii) An observation in schools showed that 15% of pit latrines were extremely filthy, 10.5% had no doors and 13.7% were unusable because they were either full or collapsed.
Mwai et al. [30] / 2016	To assess KAP on the control and prevention of schistosomiasis infection	Cross sectional study utilizing a mixed method approach	Community members aged 18 years and above (12 FGDs and 465 respondents) / Mwea Kirinyaga, Kenya	Sociodemographic variables: Awareness on schistosomiasis was significantly associated with age, educational levels. Health workers were cited as the main source of information. Knowledge: (i) Awareness was high (92.9%), (ii) only 30% had adequate knowledge on symptoms and 41% on transmission,(iii) 34.5% knew about prevention strategies *Misconception*: (i) Schistosomiasis symptoms were associated with HIV and those infected were stigmatised. (ii) Washing hands before eating and applying jelly oil before entering the rice paddies were also believed to be effective prevention strategies. Attitudes: (i) 58.71 perceived schistosomiasis to be common in the area. (ii) Treatment was perceived to be costly therefore some people preferred using herbs. Practices: Respondents acknowledged risky practices such as open air defaecation and not washing hands. Most residents did not have proper pit latrines; they were often full and unusable. Only 49.25% knew about the existence of community intervention programmes.
Musuva et al. [31] / 2014	To evaluate intervention strategies, community knowledge, attitudes, and practices on schistosomiasis in an effort to improve intervention strategies	Qualitative survey using 32 FGDs	237 Community members aged between 18 and 60 years/ Nyanza province in Kenya	Knowledge: Respondents reported having heard of schistosomiasis mainly though schools but also posters, radio and community gatherings. However, many lacked comprehensive knowledge on schistosomiasis. *Misconception:* Schistosomiasis was perceived as a sign of promiscuity. Respondents mistook it for STI’s (syphilis)*.* Respondents also believed that schistosomiasis was contracted through drinking dirty water, eating uncooked or contaminated food. Attitudes: Treatment was widely perceived as too expensive and therefore avoided. Practices: Respondents felt health facilities were far, expensive, had long queues and sometimes no drugs thus they resorted to spiritual interventions, herbal treatments and medicine shops
Onyeneho et al. [32]/2010	To assess the knowledge, attitude/perception and practices of the people in Oshimili South and Ndokwa Northeast Local Government Areas of Delta State in Nigeria	Cross-sectional study using a uniform set of structured interview schedule administered by trained field assistants.	400 randomly selected persons aged > or =15 years/Delta State in Nigeria	Knowledge: One-third of the participants were aware of the schistosomiasis. *Misconceptions:* A majority perceived schistosomiasis to be caused by witchcraft and sexual or body contact with infected persons. Infection was also considered a normal growing process. Practices: (i) Treatment was often not sought because of the belief that there is no effective cure for schistosomiasis since it reoccurs after treatment. (ii) Swimming in rivers was a common activity among all participants, irrespective of sex and age.
Odhiambo et al. [48] / 2014	To assess community awareness on existence, signs and symptoms, causes, transmission, control and risk factors for contracting schistosomiasis as well as attitudes, health seeking behaviour and environmental antecedents that affect its control	Cross-sectional, descriptive assessment that employed qualitative methods, including focus group discussions (FGDs) and key informant interviews (KIIs).	Eight focus group discussions among adult community members and eight key informant interviews with opinion leaders/Kisumu City, Western Kenya	Knowledge: Knowledge of signs and symptoms, prevention, transmission and control of schistosomiasis was poor at the beginning of MDA program. People reflected a poor understanding of preventive chemotherapy. *Misconceptions:* During MDA, Community Health Workers (CHW) were thought to be administering family planning pills thus some people rejected the drugs and chased CHW from their compounds Attitudes: Schistosomiasis was not perceived as a serious disease due to poor knowledge of signs and symptoms. There was a general belief that transmission was unpreventable. Some doubted the efficacy of drugs. Practices: (i) Poor sanitary conditions and practices were widely reported by CHW. (ii) There was poor reception of drugs due to fear of side effects and misconceptions however during the third year of MDA, there was notable improvement
Rassi et al. [33]/2016	To determine knowledge, attitudes and practices relating to schistosomiasis	A representative cross-sectional household survey using a structured questionnaire	Community members from 791 households/ Nampula Province, Mozambique	Socio-demographics factors: Knowledge of schistosomiasis was associated with being male and educated. Knowledge: (i) 91% were aware of schistosomiasis and such information was from relatives, neighbours or friends. (ii) 57% had no knowledge of causes; (iii) only 26% cited correct transmission routes and preventive practices 13%. (iv) 70% were able to mention at least two correct symptoms of the disease. *Misconceptions*: (i) Most people 81%, believed schistosomiasis was transmitted through unprotected sex, (ii) 16% stated that it was hereditary or acquired during pregnancy or birth. (iii) Some believed it was acquired though drinking contaminated water. Practice: Almost half stated that they did not protect themselves and their households from the disease.
Salawu and Odaibo [41]/2016	To assess the impact of knowledge, attitudes and sociodemographic factors on schistosomiasis burden in pregnant women of rural communities of Nigeria.	A cross sectional field study using a semi-structured questionnaire	237 Pregnant women/ Ogun state Nigeria	Socio-demographic factors: Schistosomiasis infection was associated with educational level, occupation type and religion. Knowledge: Awareness on schistosomiasis was very low (34%) and less than 10% knew about the causes of schistosomiasis. *Misconceptions*: 80% of participants believed schistosomiasis is contracted by urinating at junctions or where a dog once urinated Practices: Multipurpose water usage pattern strongly predisposed the women to infection (OR 4.31, CI 2.17–8.57). Over 80% of the population visited the river either daily or weekly.
Tuhebwe et al. [42]/2015	To assess the uptake of MDA and associated factors	Cross sectional study utilizing a mixed method approach	Adults (615 respondents aged 18 years and above) in Koome Islands, Central Uganda	Sociodemographic: Uptake of praziquantel was associated with age, occupational status and the level of education Knowledge: There was inadequate knowledge about schistosomiasis transmission and prevention and these were associated with low MDA uptake. Attitude: People felt the tablets were too big, had a bad taste and they feared the side effects Practice: Long waiting time were reported barriers to MDA uptake however respondents who were knowledgeable about schistosomiasis transmission and prevention (adjusted odds ratio [AOR] 1.85, 95% CI 1.22–2.81) and reported to have received health education from the health personnel (AOR 5.95, 95% CI 3.67–9.65) were more receptive of drugs.
Yirenya-Tawiah et al. [49]/2011	To show the importance of schistosomiasis among adult populations in the Volta Basin of Ghana.	Cross-sectional survey using a structured questionnaire	A total of 3301 study subjects from 30 rural riparian communities on the Afram and Lower Volta Basin of Ghana.	Knowledge: Knowledge was significantly associated with the male status and location. (ii) 99.4% males and 88.7% females were aware of schistosomiasis as a waterborne disease, (ii) over 60% of the respondents correctly stated the cause of infection however, 36.5% of men and 22.2% of women did not know the source of infection; (iii) only 35.4% of males and 24.7% of females had knowledge on prevention. Practice: (i) 38.5% of males and 39.5% of females showed symptoms during the study period reported to have done nothing about their health condition, (ii) 23.3% of males and 29.7% of females were taking self-medication and (iii) only 23.3% of males and 29.7% of females visited a health facility.
Yirenya-Tawiah et al. [51] / 2016	To determine urogenital schistosomiasis awareness in terms of its scope and signs and symptoms	Mixed method study using a structured questionnaire; 24 focus group discussions (FGDs) were also conducted	2585 respondents aged 15–49 years from 30 riparian communities/ Endemic communities in Ghana	Socio-demographic factors: Males were more knowledgeable than females, 14.5% and 7.2% (*p* = 0.001), respectively. Knowledge: 99.4% of male respondents and 88.7% of female respondents were aware of schistosomiasis as a waterborne disease. Only 207 out of 1096 subjects (18.9%) knew that schistosomiasis can have reproductive health implications and only 12.3% of respondents knew that urogenital schistosomiasis could facilitate the acquisition of HIV.
Omedo et al. [47]/2012	To determine the Community Health Workers’ Experiences and Perspectives on Mass Drug Administration for Schistosomiasis Control in Western Kenya: The SCORE Project	Qualitative study using unstructured open-ended group discussions	65 CHWs were interviewed from the eight districts/ Western Kenya	Attitude: Community Health Workers (CHWs) reported that people had negative attitudes towards the MDA due to lack of media awareness of such an intervention and some were not comfortable with being treated by non-professionals. *Misconception:* CHWs reported that some residents rejected the drugs due to a belief that they could cause cancer, some believed they were for HIV treatment and some thought the drugs were meant to kill them. Practice: Some people refused praziquantel because they preferred taking the drug for treatment and not for prevention. The lack of food negatively affected residents’ reception of the drug.
Omedo et al. [34]/2014	To evaluate the impact of a health communication campaign for schistosomiasis in Kisumu West, Kenya: the SCORE Project	Qualitative study using FGDs	53 community health care workers/ Kisumu West, Kenya	Knowledge: Media awareness before MDA increased knowledge on schistosomiasis control and side effects which stimulated increased acceptance and demand for the drug. *Misconception:* Community Health Workers reported that some people thought praziquantel tablets were family planning pills Practice: (i) Community Health Workers reported improved work output and compliance compared to baseline.(ii) Community Health Workers perceived radios and as more effective than community gatherings as means for sensitising the community. (iii) The involvement of stakeholders in mass media campaign process was also said have been beneficial.
Mwanga and Lwambo [43] / 2013	To determine the pre- and post-intervention perceptions and water contact behaviour related to schistosomiasis in north-western Tanzania	Data was from post-intervention knowledge, attitudes and practices (KAP) questionnaire surveys conducted between 2008 and 2010	157 community members aged 15 years and above in north-western Tanzania.	Knowledge: There was a significant increase in respondents’ knowledge of the cause, transmission, symptoms and health consequences of schistosomiasis after the intervention. Practice: The frequency, duration and timing of water contacts also decreased significantly after the intervention. Reported behaviour was found to be congruous with the actual (observed) behaviour.
Studies on care givers KAP n = (4)				
Ng’weng’weta and Tarimo [35]/2016	To determine the magnitude of S. haematobium and factors associated with exposure of preschool children in Kigogo ward, Kindoni district, Dar es Salaam	Quantitative cross sectional study	A total of 408 caregivers and 424 pupils/ Kinondoni municipality, Dar es Salam, Tanzania 2016	Socio-demographic factors: Marital status was the only demographic variable significantly associated with knowledge on schistosomiasis. Knowledge: Awareness was 91.7% among care givers. The level of comprehensive knowledge was 83.6% on the mode of transmission, symptoms, treatment and preventive measures, all encompassing. Attitudes: 76% did not consider schistosomiasis a health problem yet above 90% perceived their water practices to be risky and predisposing their children to infection Practice: The high level of knowledge (83.6%) in this study was said to be a reflection of ongoing preventive chemotherapy campaigns
Ekpo et al. [36]/2010	To determine the prevalence and intensity of urinary schistosomiasis in pre-school children between the ages of 1–6 years	Qualitative survey, using 3 FGDs among adult males, adult females and pre-school children aged 4–6 years.	Care givers and Preschool children aged 1–6 / Ilewo-Orile Nigeria.	Knowledge: knowledge on transmission and treatment was poor Attitudes: Schistosomiasis was not perceived as a serious disease. *Misconceptions:* Schistosomiasis was considered a sign of virility and maturity Practices: (i) Care givers were seen during the study exposing preschool children to infection by taking them along to rivers for bathing and washing. (ii) Older children were visiting streams on their own for washing clothes, fetching water, bathing and swimming.
Ekpo et al. [46]/2012	To determine the prevalence and intensity of Schistosoma haematobium infection in preschool children aged below 6 years in two rural communities	Qualitative study using FGDs among community members	Care givers and Preschool children aged 1–6/ Ijebu East, South-western Nigeria.	Knowledge: Care givers were aware that fresh water bodies could cause schistosomiasis but did not know the mode of transmission *Misconceptions:* Most respondents’ believed drinking unclean water was the cause for schistosomiasis. Practice: (i) Community members revealed that streams were their only source of water for washing, bathing and cooking. (ii) Preschool children aged 3–6 years were observed bathing and washing in streams whereas younger preschool children were seen accompanying their mothers to streams.
Moyo et al. [50]/2016	To determine the prevalence of and risk factors for schistosomiasis among a group of preschool children in Malawi.	Cross-sectional study using a structured questionnaire	Pre-school children, aged between 6 and 60 months and caregivers/ Malengachanzi, Nkhotakota District, Malawi	Knowledge: The levels of knowledge on causation, prevention and treatment were 71%, 88%, and 80%, respectively. Practice: (i) Caregivers reported that their children accompany them to rivers/streams when conducting domestic chores. (ii) Male preschool children were said to independently play in streams along with their older peers.
School based KAP studies (*n* = 5)				
Wolmarans and De Kock [44] / 2009	To determine the influence of health education on the prevalence, intensity and morbidity of Schistosoma haematobium infections in children over a two-year period in the Limpopo Province, South Africa	Experimental study over a 2 year period, 67children in the experimental group and 179 in control groups	Schoolchildren between the ages of 4 and 14 in Mamitwa Village Limpopo Province	Knowledge: (i) 30% indicated that they had no knowledge of schistosomiasis and 43% regarded their families as main sources of information. (ii) 0% could associate the transmission of schistosomiasis with the indiscriminate passage of contaminate excreta in natural water bodies. Practice: (i) 98% indicated that they did not make use of house taps. (ii) 76% paid visits to the local river or dam as the main source of recreation, bathing and washing of clothes. (iii) Only 33% of the school children indicated that their houses were equipped with toilets.
Maseko et al. [37] / 2016	The study aimed to assess the KAPs of schoolchildren on schistosomiasis, and to identify practices that support or hinder the progress of schistosomiasis control	A descriptive quantitative cross-sectional survey using a structured questionnaire	146 Primary school children in Siphofaneni Swaziland	Sociodemographic variables: Knowledge was correlated with predictors such as male sex, always urinating in water, and always using river water for domestic practices. Knowledge: (i) 97.3% had heard about schistosomiasis. (ii) 74% knew the signs and symptoms of urinary schistosomiasis however, (iii) only 0.7% knew signs and symptoms of intestinal schistosomiasis. (iv) 52.7% participants had knowledge of preventive measures *Misconceptions:* Misconceptions noted were said to be related to prevention and treatment Attitudes: The mean score of schistosomiasis attitudes was 86.8%. Practice: 78.8% reported being engaged in risky water practices and 65.1% was the mean score for practices.
Chaula and Tarimo [45] / 2014	To assess the impact of the two rounds of MDA on prevalence and intensity of Schistosoma haemamtobium and the impact of MDA campaigns on knowledge of urinary schistosomiasis, safe water use and contact with potentially unsafe water bodies.	A quantitative cross-sectional study. A structured questionnaire was used to collect data	488 schoolchildren, Bahi district in central Tanzania.	Practice: Uptake of MDA was 39.5% in 2011 and 43.6% in 2012. MDA campaigns had significant impact on knowledge of the disease (*p* = 0.02) and borderline impact on safe water use (*p* = 0.04) but had no impact on avoidance of contact with unsafe water bodies (*p* = 0.06)
Mazigo et al. [38] / 2010	To determine the prevalence of *Schistosoma mansoni*, knowledge, perceptions and preventative practices of school children towards	Cross sectional study using a structured questionnaire	200 randomly selected school children/ Sengerema district, Tanzania.	Sociodemographic variables: Knowledge significantly increased with age Knowledge: (i) About 87.5% of the respondents reported to have heard about schistosomiasis and the main source of information were schools. (ii) Only 40.5% of the respondents associated schistosomiasis with water contact and (iii) 39.5% accurately quoted symptoms. (iv) 34.5% respondents mentioned correct control strategies against schistosomiasis. Practice: (i) 84% of the children reported going to the lake and 68% reported to participate in paddy cultivation. (ii) Most of respondents (96.5%) reported the use of toilets. (iii) A majority (82%) of the respondents reported that they had participated in previous MDA.
Person et al. [56] / 2016	To better understand community knowledge, perceptions, and practices associated with schistosomiasis among school-aged children on Unguja and Pemba islands	Qualitative study involving 35 children’s discussion groups, 41 in-depth interviews with parents and teachers, and 5 focus group discussions with community members	School children, parents, teachers and community leaders /Zanzibar, United Republic of Tanzania	Knowledge: (i) there was poor knowledge on disease transmission, (ii) lack of understanding on severity of disease-associated consequences, (iii) and lack of alternative options for water related activities of daily living and recreational play Practices: School-aged children were regularly exposed to contaminated natural, open freshwater bodies through practicing daily recreational and domestic activities

### Knowledge and awareness about schistosomiasis

Current findings indicated that schistosomiasis awareness ranged between 75 and 98% [8, 28–38]. However, comprehensive knowledge on the signs and symptoms, transmission, prevention and treatment was observed to be low. Other studies further showed that participants confused the prevention and transmission of schistosomiasis with that of soil transmitted helminths (STH). For instance, several studies [28, 31, 38, 39] reported that the majority of participants suggested that schistosomiasis was transmitted through drinking contaminated water or eating contaminated food. Nine studies evaluated the impact of the health education component of MDA programs and reported increase in participants’ knowledge, attitudes and practices related to schistosomiasis [17, 31, 34, 40–45].

### Attitudes, misconceptions, beliefs and perceptions

The results from the review show that about 60% (16/27) of the studies reported misconceptions regarding schistosomiasis prevention and control. The misconceptions that schistosomiasis was caused by drinking unclean water and eating contaminated food were reported by most studies across Sub-Saharan Africa [16, 28, 31, 33, 39, 46]. However, in Kenya, participants believed that schistosomiasis is caused by HIV [30, 47]. Furthermore, some participants mistook syphilis for schistosomiasis [31]. On the other hand, participants in Nigeria and Mozambique [32, 33] thought that schistosomiasis was transmitted through unprotected sex and sharing of toilets with infected people infected with schistosomiasis [29]. Urinating at T-junctions of roads [17, 41] and witchcraft [32] were perceived as causes of schistosomiasis in Nigeria. Results from Uganda, Nigeria, and Mozambique suggest that the disease is believed to be hereditary, acquired during pregnancy or birth and thus considered a natural disease that could be outgrown with time [28, 32, 33, 36]. Additionally, the current study indicates that praziquantel was mistaken for family planning tablets strategically used by MDA programs to prevent people from having many children [34]. The drug was also perceived to be expensive [17, 30, 31], dangerous due to side effects [28, 40, 42] and the efficacy was questioned by some respondents due to the re-occurrence of symptoms after treatment [32, 48]. These reasons contributed to poor health seeking behaviour with some respondents eventually resorting to the use of traditional medicine and spiritual interventions [28, 31].

### Practices on schistosomiasis prevention and treatment

The results from the current study indicate that water related practices such as harvesting hippo grass, fishing, washing clothes, washing utensils, bathing and fetching water from rivers or streams and open air defaecation were observed to be risky behaviour practices that enhanced disease transmission [28–33, 37–39, 44, 46, 49, 50]. Results further indicate that the use of community gatherings, radios, posters and the involvement of local stake holders in schistosomiasis prevention campaigns were that most preferred health promotion strategies [34]. According to Adeneye et al. [17], community directed systems were more successful compared to primary health care centred and school based approaches.

### Association between awareness, knowledge, attitudes and behaviour

In this study, high levels of awareness were not congruent with high knowledge levels of the disease. Participants and respondents with higher levels of knowledge were more likely to present with better attitudes towards schistosomiasis prevention and treatment. Knowledgeable participants were also more receptive of treatment compared to those with poor schistosomiasis related knowledge. Misconceptions are said to have contributed to poor health seeking behaviour with some respondents eventually resorting to the use of traditional medicine and spiritual interventions [28, 31].

### Community, care givers and school based studies

Results from this study show limited KAP across countries. However, studies from Tanzania [35] and Malawi [50] reported adequate knowledge shown by composite scores of 83.6 and 79.7%, respectively. In Tanzania, the high level of knowledge was said to be a result of a MDA campaign which was ongoing at the time of the study. The exposure to freshwater bodies varied according to the study population. For instance, community members and care givers mainly visited streams and rivers for washing and bathing their young children while school children mainly contacted freshwater bodies during recreational activities such as swimming.

### Sociodemographic factors related to schistosomiasis KAP

Results from the reviewed studies suggest that awareness and knowledge of schistosomiasis was poor among women and uneducated members of the community [16, 28–30, 33, 39, 41, 42, 49, 51]. In contrast, other studies observed that participants below the age of 20 were more knowledgeable and aware about schistosomiasis [29, 30, 37, 42]. With regards to studies conducted among care givers in Tanzania, Ng’weng’weta and Tarimo [35] found that married women had significantly higher levels of schistosomiasis related knowledge compared to unmarried women. Nigerian caregivers were observed washing in the company of younger preschool children while those aged 3 to 6 years were found to be more adventurous, bathing and swimming independently [36, 46] thus increasing their exposure to risk of schistosoma contraction. Such findings are in consonant with Moyo and Changadeya [50] who concluded that in Malawi, 4 year old preschool children were 5.26 times at higher risk of contracting urogenital schistosomiasis infection compared to 2 year old preschool children. Results from other studies also indicated that religion [41]; schistosomiasis treatment history [5, 30] and occupation [28, 29, 39, 41, 42] were associated with better knowledge and practices.

## Discussions

This review applied a systematic approach to assess the knowledge, attitudes and practices on schistosomiasis in Sub-Saharan Africa. The study encompassed KAP studies conducted among community members, school children and care givers of preschool children. Such a combination of studies from diverse settings embody the diverse nature of findings, considering that Sub-Saharan Africa is reasonably heterogeneous in terms of sociocultural, economic and demographic profiles which may impact on schistosomiasis control. The study built on the existing literature highlighting salient socio-cultural influences that form barriers towards the prevention and control of schistosomiasis.

### Factors hindering schistosomiasis prevention and control

#### The influence of knowledge levels

The results obtained from the studies reviewed indicate lack of comprehensive knowledge relating to schistosomiasis transmission, prevention and control. This is unfortunate considering that the proficiency of correct, comprehensive knowledge in promoting healthy attitudes and protective behaviour has been long established [52, 53]. Adoka et al. [39] observed that only 3% of respondents in Lake Victoria basin could relate schistosomiasis to snails. The reduction in the levels of knowledge among the respondents may be attributed to reduced community education [54]. According to Inobaya et al. [15], community based education may lead to behavioural change like avoidance of contact with infested water bodies and contamination of the environment with faeces. However, for behavioural change to be feasible, health education should be coupled with the provision of safer water sources and basic sanitation [16, 29]. Other authors argue that knowledge does not directly translate into behaviour change but it promotes positive attitudes towards the behaviour in question [15, 19]. Notwithstanding this, the role of comprehensive knowledge as a behaviour change agent is well established.

Previous studies have observed that lower levels of knowledge on schistosomiasis and other NTDs is often accompanied by increased levels of misconceptions which may lead to poor prevention practices [34, 42, 45]. Then current study suggests that preventive chemotherapy programmes are likely to be successful if they incorporate a comprehensive health education component on schistosomiasis prevention and control [17, 28, 40]. Another issue raised by previous authors is the lack of a standard definition of comprehensive schistosomiasis related knowledge across countries [10, 52]. In this review we observed variation in terms of the scope of health education messages. For instance, very few studies reported on information pertaining to the life cycle and types of schistosomiasis [28, 39]. We have further observed that participants with higher knowledge were more likely to adopt protective behaviour towards schistosomiasis infection [31, 50, 55]. Such findings propose the use of standardised context specific training manuals appropriately designed to suit school children and community members in order to limit variation in health education messages.

#### Socio-demographic determinants

Age, gender and level of education were widely shown to have an impact on schistosomiasis knowledge and practices. The impact of these social determinants in addition to socioeconomic factors on health promotion interventions at individual, community and organizational levels have been reported [14, 55]. The current study observed that uneducated women were more vulnerable to schistosomiasis infection due to their daily water related house chores coupled by poor knowledge on preventive and control measures. In a study conducted by Ng’weng’weta and Tarimo [35], marital status was associated with knowledge indicating that married couples were more likely to share health related knowledge. Older community members were more prone to poor schistosomiasis related knowledge compared to school going children [33, 36, 46, 56]. Nevertheless, risky water contact practices such as swimming were more common among school going children [16, 37, 45, 48]. These findings suggest that an understanding of the socio-demographic and socio-economic profiles of communities may contribute to implementation of effective programmes aimed at controlling schistosomiasis.

Only 12 of all the studies reviewed reported on the influence of sociodemographic variables on schistosomiasis knowledge. Eight of the 12 studies consistently reported a significantly higher level of knowledge among males compared to females. In a study conducted by Ng’weng’weta and Tarimo (2016) however marital status was the only sociodemographic variable significantly associated with knowledge. In a study conducted by Mazigo et al. [2010] males were more exposed to risky water practices such as fishing compared to females and studies conducted in Kenya and Nigeria discovered that fishermen and farmers had better knowledge on schistosomiasis [28, 39]. Furthermore, Tuhebwe et al. [42] found that being a fisherman was positively associated with the increased uptake of Praziquantel during their mass drug administration.

#### Attitudes and misconceptions

Acquisition and dissemination of the right information is key in the reduction of schistosomiasis infection risks. Some authors [28, 53], suggested that the right information equips people with knowledge on the schistosomiasis transmission cycle. It also enables them to adopt safer water and sanitary practices. The reviewed studies suggest that in rural communities, health related messages are shared among friends and neighbours. These massages, if not explained properly, may lead to misconceptions and promote superstitious beliefs as observed by several authors [16, 17, 40, 49]. Such beliefs may affect the utilization of health services leading to delayed diagnosis and treatment [14]. Sixty percent (60%) of the studies reviewed indicated that schistosomiasis was a sexually transmitted infection [29, 30, 32, 33, 47]. Such findings are consistent with previously reported findings [1, 5]. These studies concluded that misconceptions regarding schistosomiasis and STH transmission were rampant among people living in endemic areas. The confusion regarding information on the mode of transmission relating to schistosomiasis and STH suggests the lack of integrative approaches towards schistosomiasis and STH programming. Previous findings have highlighted the importance of addressing social and environmental issues in order to achieve effective comprehensive control programs for helminthic infections [57].

#### Practices associated with risks of infection

Our study observed that methods employed by caregivers may predispose their children to schistosomiasis infection [13, 36, 50]. This is because water contacts activities such as bathing and washing may be performed in the company of infants and preschool children [36, 46]. The vulnerability of and preschool children is substantiated by evidence from studies conducted in Mali [58], Niger [13], Sudan [59], Uganda [60] and Zimbabwe [61] where high prevalence of schistosomiasis among preschool children ranged from 18 to 63%. The importance of adopting sound strategies that empower community members to take relatively simple measures to prevent disease, protect their health and adhere to treatment is well described in literature [62, 63]. However, the work conducted in Kenya [30] and South Africa [44] suggest that the effect of poor knowledge on disease prevention practices can be mitigated by the presence of basic environmental and health services, such as the availability of safer sources of water supply and adequate sanitation. Studies conducted in Nigeria, Malawi and Swaziland [13, 37, 50] reflected that knowledge alone may not be enough to influence healthy practices. The authors further noted the persistence of risky sanitary and water related practices among care givers and school children with comprehensive knowledge on schistosomiasis. This suggests the need for an integrative approach for effective prevention and control of schistosomiasis. This can be achieved through integrated implementation of MDA with proper sanitation education programmes, provision of safe water supply and health education for effective schistosomiasis control.

### Towards schistosomiasis prevention and control

The current findings highlight the significance of health education as a key component of the schistosomiasis control programs [31, 51, 55]. This is because such programs are said to improve awareness, knowledge and uptake of preventive chemotherapy [17, 31, 34, 40, 42, 45, 47, 48], thus increasing the success of MDA programmes. The major limitation of schistosomiasis control programs in endemic countries is the inadequate coverage and sustainability of programmes which is not only due to poverty but also to social, cultural, and political forces that remain understudied [5]. Previous epidemiological studies have advocated for the adaptation of prevention and control strategies to people’s livelihoods in order to achieve high levels of MDA uptake and adoption of protective behaviours towards schistosomiasis prevention and control [1, 55]. It is therefore imperative to consider the influence of social, cultural and cognitively rooted influences on health risk behaviours in schistosomiasis prevention and control strategies.

### Limitations

Limitations of the study could be related to the lack of a standard definition for awareness, knowledge, attitudes and practices and the skewed focus on articles published in English. The review however, attempted to give a comprehensive description of studies conducted among community members, caregivers and school children even though few KAP studies were found under the two latter categories. Articles with richer descriptions may have been over-represented in the study only because they provided the required information. The review does not present quantifications to characterize the literature thus a significant portion of the decision-making, abstraction and interpretation is subjective. We therefore convened regular group discussions to evaluate our understanding of the subject throughout the review process.

## Conclusion

In conclusion, the study suggests the need for changes in knowledge, attitudes and practices of people in relation to schistosomiasis prevention and control. Contextual and standardised training tools tailored for community and school based schistosomiasis control programs are of great necessity. Studies focusing on schistosomiasis among preschool children should not overlook the role that can be played by caregivers in protecting children from infection provided they are adequately empowered to do so. This review emphasises on the importance of incorporating a health education component to both community and school based MDA programs. Further research focused on evaluating the impact of existing control programs on the KAP and disease burden among beneficiaries of such programs is imperative.

## Additional files

Additional files 1: Quality Assessment of individaul studies. This is a tool that was used for critical appraisal of the studies included in the review. This tool was modified from the initially developed by Downes and Brennan [27] (DOCX 23 kb)

## Abbreviations

HIV: Human Immunodeficiency Virus
KAP: Knowledge, Attitudes and Practices
MDA: Mass Drug Administration
NTD: Neglected Tropical Diseases
PRISMA: Preferred Reporting Items for Systematic Reviews and Meta-Analyses
SEED: Supply–Enabling Environment–Demand
STH: Soil Transmitted Helminths
STI: Sexually Transmitted Infections
WHA: World Health Assembly
WHO: World Health Organization
