# Supplementary material for: Knowledge, attitudes and practices on Schistosomiasis in sub-Saharan Africa: a systematic review
Source: BMC Infect Dis. 2018 Jan 18;18:46. doi: 10.1186/s12879-017-2923-6 (PMC5773048; doi:10.1186/s12879-017-2923-6)
Supplement: Supplementary file 1 — Quality Assessment of individaul studies. This is a tool that was used for critical appraisal of the studies included in the review. This tool was modified from the initially developed by Downes and Brennan [27] (DOCX 23 kb) [file 12879_2017_2923_MOESM1_ESM.docx]

| Quality appraisal was assessed using a 10-point scoring system quality assessment tool for cross-sectional studies  The total quality score varied between 0 and 10 where 1-4 = (Low); 5-7 = (Moderate) and 8-10= (High) | | | | | | | | | | | |
| --- | --- | --- | --- | --- | --- | --- | --- | --- | --- | --- | --- |
| Introduction | | **Methods** | | | | | **Results** | | **Discussions** | | **TOTAL** |
| Author/  Year | (1) clear definition of objectives/ aims | (2) study design appropriate for the stated aims | (3) sample size justified | (4) target population clearly defined (appropriate population base/unbiased sampling) | (5) risk factor and outcome variables measured correctly using instruments that had been trialled, piloted or published previously | (6) methods (including statistical methods) sufficiently described to enable them to be repeated | (7) results for analysis described in the methods, presented | (8) authors discussions and conclusions justified by results | (9) limitations of the study discussed | (10) ethical approval or consent of participants attained | Scores  (0-10) |
| Adoka et al. [20] / 2014 | 1 | 1 | 0 | 1 | 1 | 1 | 0 | 1 | 0 | 1 | 7 |
| Anguza et al. [21] / 2007 | 1 | 1 | 1 | 1 | 1 | 0 | 0 | 1 | 0 | 1 | 7 |
| Adeneye et al. [17] / 2007 | 1 | 1 | 0 | 1 | 1 | 1 | 0 | 1 | 0 | 0 | 6 |
| Dawaki et al. [22] / 2015 | 1 | 1 | 0 | 1 | 1 | 1 | 0 | 1 | 0 | 0 | 6 |
| Fleming et al. [23] / 2009 | 1 | 1 | 1 | 1 | 1 | 0 | 0 | 1 | 1 | 0 | 7 |
| Kabatereine et al. [16] / 2014 | 1 | 1 | 0 | 1 | 1 | 1 | 0 | 1 | 0 | 1 | 7 |
| Mwai et al. [24] / 2016 | 1 | 1 | 1 | 1 | 1 | 1 | 0 | 1 | 0 | 0 | 7 |
| Musuva et al. [25] / 2014 | 1 | 1 | 0 | 1 | 1 | 1 | 0 | 1 | 0 | 1 | 7 |
| Onyeneho et al. [26] / 2010 | 1 | 1 | 0 | 1 | 1 | 0 | 0 | 1 | 0 | 1 | 6 |
| Odhiambo et al. [27] / 2014 | 1 | 1 | 0 | 1 | 1 | 1 | 0 | 1 | 0 | 0 | 6 |
| Rassi et al. [28] / 2016 | 1 | 1 | 1 | 1 | 1 | 1 | 1 | 1 | 0 | 0 | 8 |
| Salawu and Odaibo [29] / 2016 | 1 | 1 | 1 | 1 | 1 | 1 | 0 | 1 | 0 | 1 | 8 |
| Tuhebwe et al. [30] / 2015 | 1 | 1 | 0 | 1 | 1 | 1 | 1 | 1 | 1 | 1 | 9 |
| Yirenya-Tawiah et al. [31] / 2011 | 1 | 1 | 0 | 1 | 0 | 1 | 0 | 1 | 0 | 1 | 6 |
| Yirenya-Tawiah et al. [32] / 2016 | 1 | 1 | 0 | 1 | 1 | 0 | 0 | 1 | 0 | 0 | 5 |
| Omedo et al. [33] / 2012 | 1 | 1 | 0 | 1 | 1 | 1 | 0 | 1 | 0 | 1 | 7 |
| Omedo et al. [34] / 2014 | 1 | 1 | 0 | 1 | 1 | 1 | 0 | 1 | 0 | 1 | 7 |
| Mwanga and Lwambo [35] / 2013 | 1 | 1 | 0 | 1 | 0 | 1 | 0 | 1 | 0 | 1 | 6 |
| Ng'weng'weta and Tarimo [36] / 2016 | 1 | 1 | 1 | 1 | 1 | 1 | 1 | 1 | 0 | 1 | 9 |
| Ekpo et al. [37] / 2010 | 1 | 1 | 1 | 1 | 0 | 1 | 1 | 1 | 0 | 1 | 8 |
| Ekpo et al. [38] / 2012 | 1 | 1 | 1 | 1 | 0 | 1 | 1 | 1 | 0 | 1 | 8 |
| Moyo et al. [39] / 2016 | 1 | 1 | 0 | 1 | 0 | 0 | 1 | 1 | 0 | 1 | 6 |
| Wolmarans and De Kock [40] / 2009 | 1 | 1 | 0 | 1 | 0 | 1 | 0 | 1 | 0 | 0 | 5 |
| Maseko et al. [41] / 2016 | 1 | 1 | 0 | 1 | 0 | 1 | 0 | 1 | 0 | 1 | 6 |
| Chaula and Tarimo [42] / 2014 | 1 | 1 | 1 | 1 | 0 | 0 | 0 | 1 | 0 | 1 | 6 |
| Mazigo et al. [43] / 2010 | 1 | 1 | 0 | 1 | 0 | 0 | 0 | 1 | 0 | 1 | 5 |
| Person et al. [44] / 2016 | 1 | 1 | 0 | 1 | 1 | 1 | 0 | 0 | 1 | 1 | 7 |
